# The taxane-based chemotherapy triplet is superior to the doublet in one to nine node-positive but not node-negative triple-negative breast cancer: results from a retrospective analysis

**DOI:** 10.7150/jca.44768

**Published:** 2020-09-23

**Authors:** Sanxing Guo, Yonggang Shi, Shuo Lu, Yujie He, Guangyi Jin, Suzhi Zhang, Xingya Li

**Affiliations:** 1 Oncology Department, The First Affiliated Hospital of Zhengzhou University, Zhengzhou, China; 2 Department of Radiotherapy, The First Affiliated Hospital of Zhengzhou University, Zhengzhou, China; 3 School of Basic Medicine, Guangdong Medical University, Shenzhen, China; 4 Rheumatology and Immunology Department, The First Affiliated Hospital of Zhengzhou University, Zhengzhou, China; 5 International Cancer Center, Shenzhen University Health Science Center, Shenzhen, China; 6 National Engineering Lab for Synthetic Biology of Medicine, Shenzhen University, Shenzhen, China; 7 Department of Pharmacy, The First Affiliated Hospital of Zhengzhou University, Zhengzhou, China

**Keywords:** triple-negative breast cancer, taxane-based regimen, chemotherapy, lymph node stage

## Abstract

**Background**: Taxane-based regimens that are frequently used in adjuvant chemotherapy in early triple-negative breast cancer (TNBC) include a three-drug regimen (TAC and AC-T) and a two-drug regimen (TA and TC). Whether pathological lymph node stage guides taxane-based de-escalating chemotherapies in TNBC in adjuvant setting is still unclear.

**Methods**: We retrospectively examined 381 patients with early TNBC over a median follow-up period of 75.9 months and compared the disease-free survival (DFS) and overall survival (OS) of patients who received adjuvant taxane-based three-drug chemotherapy and two-drug chemotherapy according to pathological lymph node stage (negative, pN0; 1-3 positive, pN1; 4-9 positive, pN2).

**Results**: In 222 pN0 patients, the taxane-based three-drug regimen was not superior to the two-drug regimen. In 159 pN1-2 patients, the taxane-based three-drug regimen significantly improved DFS (5-year DFS rate, 78.2% vs. 46.9%; log-rank *p*=0.0002) and OS (5-year OS rate, 90.7% vs. 64.3%; log-rank *p*=0.0001) compared with the two-drug regimen. In a multivariable Cox regression analysis of pN1-2 patients, after adjustment for clinical characteristics, the taxane-based three-drug regimen significantly decreased the risk of recurrence (adjusted HR, 0.37; 95% CI, 0.22 to 0.64; *p*=0.0004) and death (adjusted HR, 0.22; 95% CI, 0.10 to 0.48; *p*=0.0001) compared with the two-drug regimen.

**Conclusions**: The taxane-based chemotherapy triplet is superior to the chemotherapy doublet in patients with one to nine positive lymph nodes but not node-negative TNBC in adjuvant setting. These data suggest that pathological lymph node stage leads to de-escalating chemotherapy strategies in operable TNBC patients.

## Background

Triple-negative breast cancer (TNBC) is used to describe a subset of breast cancers and is defined as the lack of expression of estrogen receptor (ER) and progesterone receptor (PR) and the absence of human epidermal growth factor receptor 2 (HER2) overexpression and/or gene amplification 1. Although extensive efforts have been undertaken to find new therapeutic targets of TNBC based on its molecular landscape in the past decade, targeted therapy has shown limited clinical success. Patients with TNBC are still mainly dependent on conventional chemotherapy. Hence, the optimal tailoring of conventional chemotherapy should be continued to establish the most effective regimens.

Anthracyclines have been a backbone of breast cancer chemotherapy for more than three decades, with extensive data supporting their value in improving disease-free survival (DFS) and overall survival (OS) 2. Additionally, taxanes have emerged as particularly active agents against breast cancer 3. Adjuvant polychemotherapy with taxanes given concurrently or sequentially with anthracyclines has resulted in reduced recurrence and death rates compared to anthracycline-based regimens alone 4,5. Consequently, taxane-based chemotherapy regimens are now widely used as (neo)adjuvant chemotherapy for breast cancer and TNBC. These taxane-based regimens mainly include (1) a taxane, an anthracycline, and a cyclophosphamide every 3 weeks for 6 cycles (TAC); (2) an anthracycline plus a cyclophosphamide every 3 weeks for 4 cycles followed by a taxane every 3 weeks for 4 cycles (AC-T); (3) a taxane plus an anthracycline every 3 weeks for 6 cycles (TA); or (4) a taxane plus a cyclophosphamide every 3 weeks for 6 cycles (TC). In the present study, TAC and AC-T are described as the taxane-based three-drug regimen, and TA and TC are described as the taxane-based two-drug regimen.

However, there is no unified standard for selecting the taxane-based three-drug regimen or two-drug regimen for early-stage breast cancer. Recently, a large-scale randomized study comparing adjuvant cyclophosphamide and docetaxel with or without epirubicin (EC-D vs. DC) in early TOP2A-normal breast cancer showed no OS benefit from adjuvant anthracyclines, but grade 3 adverse events were reported more frequently in the three-drug regimen compared with the two-drug regimen 6. However, the anthracyclines in early breast cancer (ABC) trials performed on a large number of patients compared the DFS of the TAC and TC regimens in patients with HER2-negative early-stage breast cancer. The TAC regimen improved DFS in patients with high-risk HER2-negative breast cancer compared with the TC regimen (TAC vs. TC, 4-year invasive disease-free survival (IDFS) rate, 90.7% vs. 88.2% p=0.04) 7. These two large-scale studies showed inconsistent results when the taxane-based three-drug regimen was compared with the two-drug regimen. However, grade 3 adverse events were reported more frequently in patients treated with the three-drug regimen compared with patients treated with the two-drug regimen. In addition, Carlos H et al. reported that the taxane-based three-drug regimen was associated with a higher risk of chemotherapy-related hospitalization in patients with early-stage breast cancer compared with the TC regimen 8. Based on the different adverse event profiles, it has been a challenge to evaluate the de-escalation of the taxane-based three-drug regimen in patients with early-stage breast cancer.

The status of the axillary lymph nodes is an important prognostic factor in breast cancer 9,10. Approximately one-third of patients with TNBC are diagnosed with lymph node involvement 11. A subgroup analysis of the ABC trials showed that patients with TNBC might benefit more from the TAC regimen than from the TC regimen when the number of positive lymph nodes increases 7. The current study explored the efficacy of the taxane-based three-drug regimen and the two-drug regimen according to pathological lymph node stage and provided evidence for individualized chemotherapy in patients with early TNBC.

## Methods

### Patients and Treatments

Clinical data of patients with early-stage TNBC were retrospectively collected in the First Affiliated Hospital of Zhengzhou University from August 2007 to December 2014. Eligible patients were women aged 18 to 75 years who had undergone surgery for a unilateral operable invasive breast carcinoma (cT1-3 pN0-2 M0). Tumor sizes were limited from 1 cm to 7 cm as measured by ultrasound (more than 90%) or palpation. All patients received modified mastectomy or conservative surgery with tumor-free margins (R0) and had pathologically evaluated negative or 1-9 positive axillary lymph nodes (pN0-2) by axillary dissection (≥10 nodes examined) or sentinel lymphadenectomy (≥4 nodes examined). Patients undergoing breast conservative surgery were required to receive postoperative radiation therapy after the completion of chemotherapy. HER2 status (immunohistochemistry 1+ or in situ hybridization ratio < 2.0) and ER and PR status (< 1%) were assessed as TNBCs. HER2 expression was evaluated according to the American Society of Clinical Oncology-College of American Pathologists (ASCO-CAP) guidelines 12.

All the patients received taxane-based three-drug or two-drug adjuvant chemotherapy. The taxane-based two-drug regimen included a taxane plus an anthracycline every 3 weeks for 6 cycles (TA) and a taxane plus a cyclophosphamide every 3 weeks for 6 cycles (TC). The three-drug regimen included a taxane, an anthracycline plus a cyclophosphamide every 3 weeks for 6 cycles (TAC) and an anthracycline plus a cyclophosphamide every 3 weeks for 4 cycles followed by a taxane every 3 weeks for 4 cycles (AC-T); all patients completed the planned cycles. The patients who received neoadjuvant chemotherapy were excluded. Chemo-regimen crossover was excluded. The current study was approved by the First Affiliated Hospital of Zhengzhou University Ethics Committee. As this was a retrospective analysis, this study was exempt for informed consent.

### Outcomes for Analysis

The main outcomes analyzed were DFS and OS. DFS was defined as the time from primary diagnosis to the date of clinical relapse, a second malignant tumor (operable nonmetastatic papillary thyroid cancer was excluded), or death. OS was defined as the time from primary diagnosis to the date of death from any cause. DFS and OS in the two treatment groups were assessed by the log-rank test and stratified according to lymph node stage (N0, tumor with negative lymph nodes; N1, tumor with 1-3 positive lymph node(s); N2, tumor with 4-9 positive lymph nodes).

### Statistical Analysis

The Kaplan-Meier method was used to calculate the probability estimates of DFS and OS. The resulting curves between treatment groups were compared by using the log-rank test. A Cox proportional hazards model was used to estimate unadjusted hazard ratios (HRs) and 95% confidence intervals (CIs) for DFS and OS. Multivariate Cox proportional hazards models (adjusted for major prognostic factors, including age, tumor stage, and histopathological features) were applied to estimate the adjusted HRs and 95% CIs for DFS and OS. Associations between regimens and other characteristics were analyzed by Fisher's exact test. All analyses were two sided, and a p value less than 0.05 was considered significant, as assessed with SPSS version 21.0 (SPSS, Inc., Chicago, Illinois, USA).

## Results

### Patients

From August 2007 to December 2014, a total of 381 patients from the First Affiliated Hospital of Zhengzhou University were selected for this study. Of the 381 patients, 222 were in pN0 stage, and 159 patients were in pN1-2 stage (Table 1). Specific demographic and histopathological features and clinical treatments of the patients are shown in Table 1. Although a greater proportion of older patients received the chemotherapy doublet, no significant difference (pN0 group, *p*=0.28; pN1-2 group, *p*=0.74) between the two treatment groups was observed. Other baseline characteristics of the patients were well balanced between the two treatment groups according to lymph node status. Approximately 9.2% of patients censored with an overall follow up less than 4 years.

### Efficacy

At a median follow-up of 75.9 months, 76 primary events had occurred, including 71 patients with breast cancer relapse and 5 second primary malignancies. The first observed DFS events are summarized in Table 2. Additionally, 6 patients were diagnosed with operable nonmetastatic papillary thyroid cancer, and all patients received surgery. During the follow-up, there was no papillary thyroid cancer-related recurrence or death. In the current study, secondary papillary thyroid cancer was excluded from DFS events.

**Figure 1:**
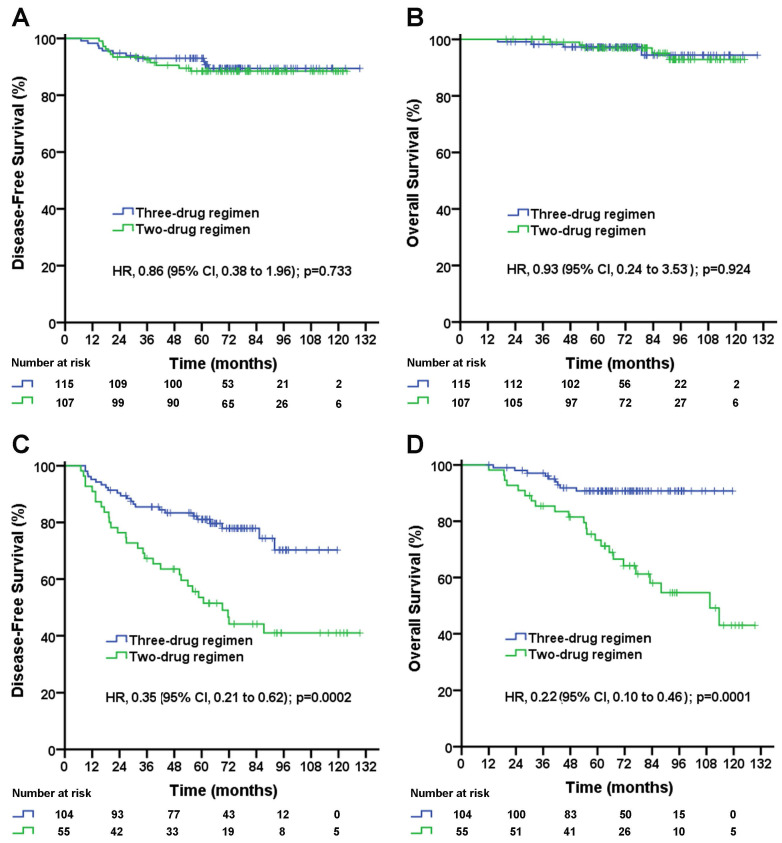
Disease-free survival (A) and overall survival (B) for triple-negative breast cancer patients with negative lymph nodes according to treatment group; disease-free survival (C) and overall survival (D) for triple-negative breast cancer patients with 1-9 positive lymph node(s) according to treatment group. The unadjusted hazard ratios for disease recurrence and death are shown. The three-drug regimen includes the TAC and AC-T regimens, and the two-drug regimen includes the TA and TC regimens. TAC= taxane, anthracycline, and cyclophosphamide. AC-T= anthracycline plus cyclophosphamide followed by a taxane. TA= taxane and anthracycline. TC= taxane and cyclophosphamide. HR=hazard ratio.

In patients with pN0 stage, the median follow-up duration was 72.2 (95% CI, 68.4 to 76.0) months in the three-drug regimen group and 84.6 (95% CI, 77.5 to 91.7) months in the two-drug regimen group. No significant difference in the DFS rate (three-drug vs. two-drug, 5-year DFS rate, 89.1% vs. 88.4%; *p*=0.733; Figure 1A) or OS rate (three-drug vs. two-drug, 5-year OS rate, 93.7% vs. 96.9%; *p*=0.924; Figure 1B) was observed between the two treatment groups.

**Figure 2:**
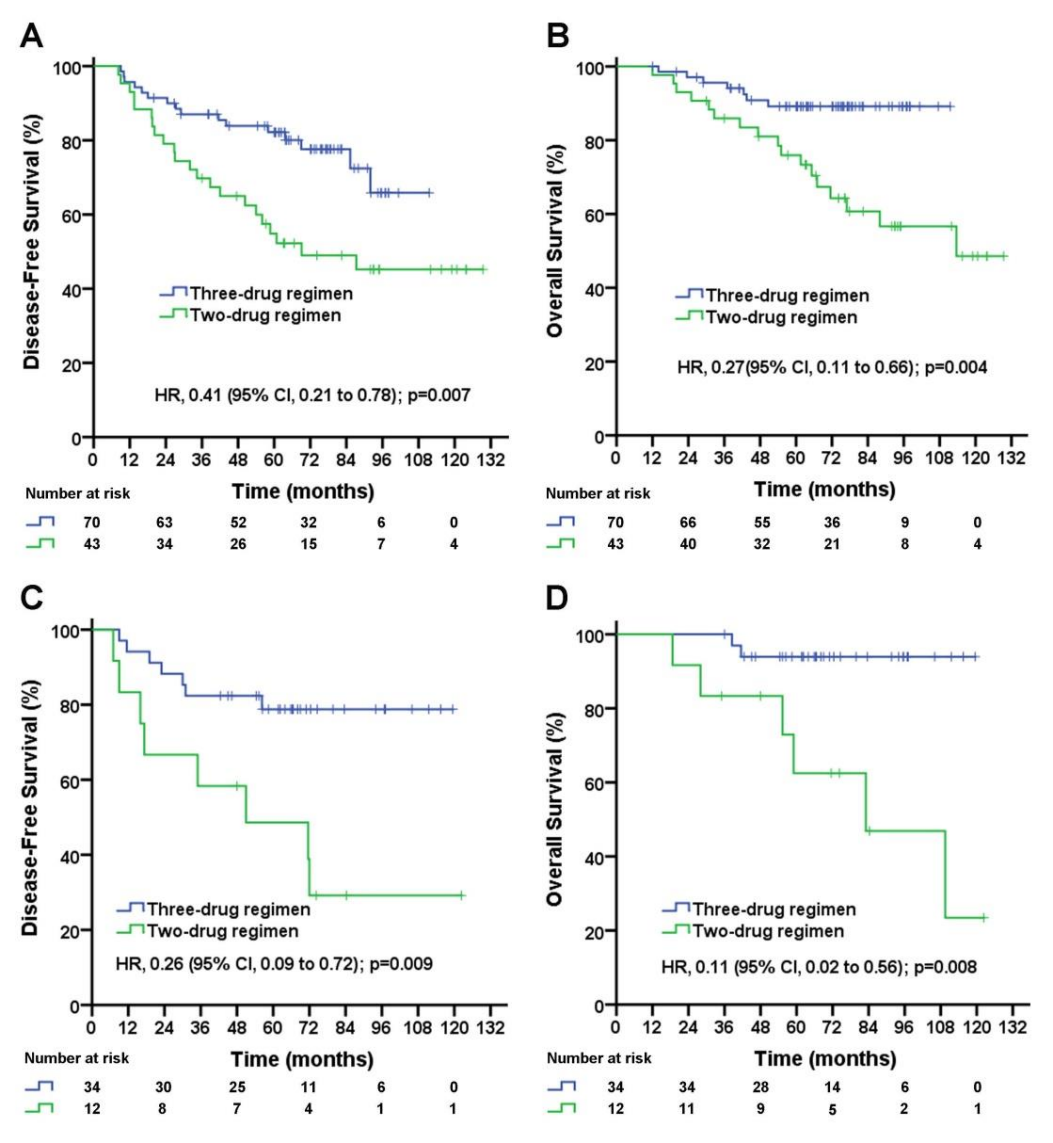
Disease-free survival (A) and overall survival (B) for triple-negative breast cancer patients with 1-3 positive lymph node(s) according to treatment group; disease-free survival (C) and overall survival (D) for triple-negative breast cancer patients with 4-9 positive lymph nodes according to treatment group. The unadjusted hazard ratios for disease recurrence and death are shown. The three-drug regimen includes the TAC and AC-T regimens, and the two-drug regimen includes the TA and TC regimens. TAC= taxane, anthracycline, and cyclophosphamide. AC-T= anthracycline plus cyclophosphamide followed by a taxane. TA= taxane and anthracycline. TC= taxane and cyclophosphamide. HR=hazard ratio.

In patients with pN1-2 stage, the median follow-up duration was 72.2 (95% CI, 66.0 to 78.4) months in the three-drug regimen group and 93.0 (95% CI, 79.6 to 106.4) months in the two-drug regimen group. The taxane-based three-drug regimen significantly improved the DFS rate (5-year DFS rate, 72.8% vs. 46.9%; unadjusted HR, 0.35; 95% CI, 0.21 to 0.62; log-rank *p*=0.0002; Figure 1C) and significantly improved the OS rate (5-year OS rate, 90.7% vs. 64.3%; unadjusted HR, 0.22; 95% CI, 0.10 to 0.46; log-rank *p*=0.0001; Figure 1D) compared with the taxane-based two-drug regimen. A subgroup analysis stratified by the number of positive lymph nodes was performed. In pN1 patients, the taxane-based three-drug regimen significantly improved DFS rate (5-year DFS rate, 78.2% vs. 49.0%; unadjusted HR, 0.41; 95% CI, 0.21 to 0.78; log-rank *p*=0.007; Figure 2A), significantly improved OS rate (5-year OS rate, 89.2% vs. 64.7%; unadjusted HR, 0.27; 95% CI, 0.11 to 0.66; log-rank *p*=0.004; Figure 2B) compared with the two-drug regimen. In pN2 patients, the taxane-based three-drug regimen significantly improved the DFS rate (5-year DFS rate, 78.8% vs. 39.5%; unadjusted HR, 0.26; 95% CI, 0.09 to 0.72; *p*=0.009; Figure 2C) and OS rate (5-year OS rate, 93.8% vs. 63.4%; unadjusted HR, 0.11; 95% CI, 0.02 to 0.56; *p*=0.008; Figure 2D) compared with the two-drug regimen.

Multivariable analyses of DFS and OS by patient subgroups (including age, chemotherapy mode, tumor stage, tumor grade and histologic type) were carried out. The three-drug regimen significantly decreased the risk of disease recurrence (adjusted HR, 0.37; 95% CI, 0.22 to 0.64; *p*=0.0004; Table 3) and death (adjusted HR, 0.22; 95% CI, 0.10 to 0.48; *p*=0.0001; Table 3) compared with the two-drug regimen in pN1-2 patients. In the subgroup analysis, patients both in pN1 group and in pN2 group benefited from the three-drug regimen compared with the two-drug regimen. And the three-drug regimen significantly decreased the risk of recurrence ( for pN1 patients, adjusted HR, 0.47; 95% CI, 0.24 to 0.90; *p*=0.023; for pN2 patients, adjusted HR, 0.26; 95% CI, 0.09 to 0.79; *p*=0.018; Table 4 and Table 5) and the risk of death (for pN1 patients, adjusted HR, 0.30; 95% CI, 0.12 to 0.74; *p*=0.009; for pN2 patients, adjusted HR, 0.10; 95% CI, 0.02 to 0.58; *p*=0.011; Table 4 and Table 5) in pN1 group and in pN2 group compared with the two-drug regimen after adjusting for clinical factors.

### Subgroup Analyses

**Figure 3:**
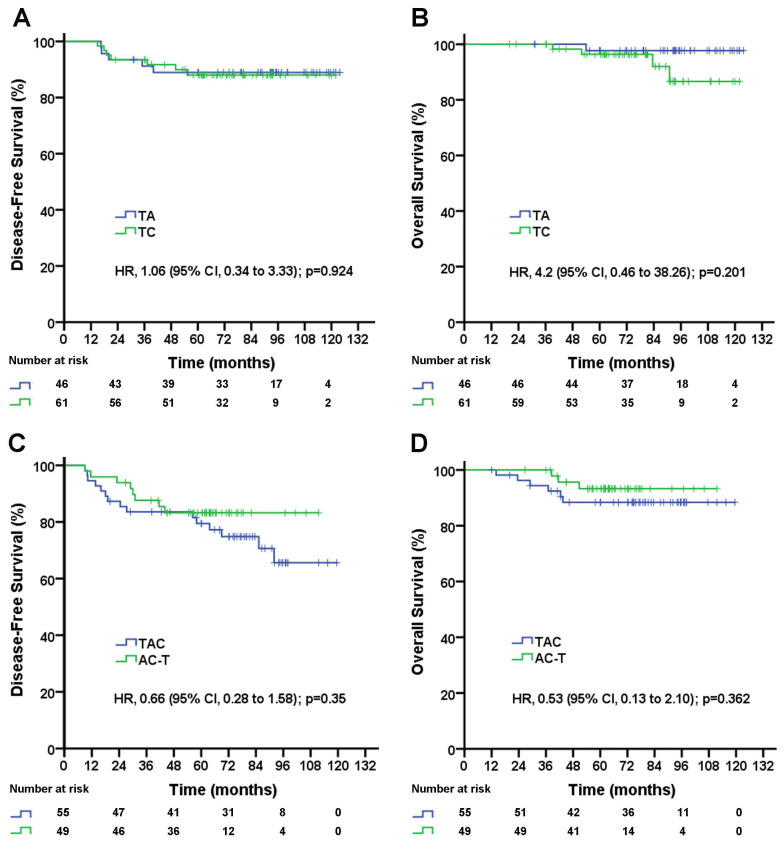
Disease-free survival (A) and overall survival (B) for triple-negative breast cancer patients with negative lymph nodes according to treatment group; disease-free survival (C) and overall survival (D) for triple-negative breast cancer patients with 1-9 positive lymph node(s) according to treatment group. TAC= taxane, anthracycline, and cyclophosphamide. AC-T= anthracycline plus cyclophosphamide followed by a taxane. TA= taxane and anthracycline. TC= taxane and cyclophosphamide. HR=hazard ratio.

As shown above, in pN1-2 patients, the three-drug regimen significantly improved DFS and OS compared with the two-drug regimen, but in pN0 patients, the three-drug regimen was not superior to the two-drug regimen for DFS and OS. The three-drug regimen and the two-drug regimen both included two different regimens. Which single regimen is better for pN1-2 patients, and which single regimen is better for pN0 patients? A subgroup analysis showed no significant difference in DFS (TA vs. TC, 5-year DFS rate, 89.0% vs. 88.0%; p=0.924; Figure 3A) or OS (TA vs. TC, 5-year OS rate, 97.7% vs. 96.3%; p=0.201; Figure 3B) between the TA and TC regimens in pN0 patients, and there was no significant difference in DFS (TAC vs. AC-T, 5-year DFS rate, 74.9% vs. 83.3%; p=0.35; Figure 3C) or OS (TAC vs. AC-T, 5-year OS rate, 88.4% vs. 93.1%; p=0.362; Figure 3D) between the TAC and AC-T regimens in pN1-2 patients. In a multivariable analysis after adjusted for clinical factors AC-T regimen did not significantly decrease risk of recurrence (adjusted HR, 0.61; 95% CI, 0.26 to 1.46; p=0.27; data not shown) and death (adjusted HR, 0.50; 95% CI, 0.13 to 2.01; p=0.329; data not shown) compared with TAC regimen in pN1-2 patients. However, sequential AC-T showed a superior trend to concurrent TAC for DFS and OS in pN1-2 TNBC patients.

## Discussion

This retrospective study suggests that pathological lymph node stage can be used to guide taxane-based chemotherapy triplet or taxane-based chemotherapy doublet regimens, which should help individualize chemotherapy in patients with operable TNBC. In pN0 patients, the taxane-based three-drug regimen was not superior to the two-drug regimen regarding DFS and OS. In pN1-2 patients, the taxane-based three-drug regimen significantly improved the DFS and OS rates compared with the two-drug regimen. According to the multivariable Cox regression analyses, the taxane-based three-drug regimen significantly decreased the risk of recurrence and death relative to the two-drug regimen irrespective of age, tumor stage, tumor grade and histologic type in pN1-2 patients.

Most patients with TNBC are still mainly dependent on cytotoxic chemotherapy, which reduces the risk of recurrence and death. Even so, approximately 20-40% of patients with early-stage TNBC develop metastatic disease 13,14. Most guidelines recommend anthracycline-based and taxane-based neo/adjuvant chemotherapy in TNBCs. The most common taxane-based regimens include TAC, AC-T, TA and TC regimens. As anthracycline and taxane-containing regimens have diversified, it has been a challenge to evaluate the de-escalation of the regimens. Here, we simply divided these four taxane-based regimens into two categories: a two-drug regimen and a three-drug regimen.

Several studies have compared the taxane-based chemotherapy triplet with the taxane-based chemotherapy doublet in HER2-negative breast cancer; although the results have been controversial, the TC regimen has shown a more favorable safety profile 7, 15, 16. Compared with non-TNBC, patients with TNBC have a higher death rate within 5 years after diagnosis than patients with other subtypes of breast cancer. Moreover, the risk of distant recurrence peaked at 3 years and declined rapidly thereafter. However, for non-TNBC, the recurrence risk remained constant over the follow-up period 13,17. In light of their different biological features, an analysis of all patients with TNBC and non-TNBC may lead to confusion and does not reflect the real results of TNBC. Hence, the current study focused on operable TNBC and found that the taxane-based chemotherapy triplet was superior to the taxane-based chemotherapy doublet in one to nine node-positive but not node-negative TNBC. The ABC trial showed that the 4-year DFS rate of the taxane-base three-drug regimen compared with the two-drug regimen was 89.5% vs. 87.0%, respectively, in lymph node-negative TNBC, 85.5% vs. 74.6%, respectively, in TNBC with 1-3 positive nodes, and 71.8% vs. 60.8%, respectively, in TNBC with ≥4 positive nodes. The benefit of the three-drug regimen was evident by the increased number of positive nodes, which is generally consistent with the present study. This finding indicates that in early TNBC, lymph node status could be used to guide chemotherapy de-escalation and escalation strategies.

A large number of studies have shown that cardiac mortality increased with anthracycline regimens, which are also associated with an increased risk for myelodysplastic syndromes and treatment-related leukemia 18,19,20. In addition, paclitaxel combined with full-dose anthracycline results in potential cardiotoxicity 21. Because of the potential cardiotoxicity resulting from the paclitaxel combination, a TA regimen has not been commonly recommended in recent years. In our study, the subgroup analysis showed similar DFS and OS rates for the TC and TA regimens (Figure 3A and 3B) in patients with lymph node-negative TNBC. Hence, TC, with similar efficacy to TA but with less toxicity, might be a better choice for patients with lymph node-negative TNBC.

In lymph node-positive (pN1-2) patients, there was a significant decrease in the risk of recurrence (adjusted HR, 0.37; 95% CI, 0.22 to 0.64; p=0.0004; Table 3) and death (adjusted HR, 0.22; 95% CI, 0.10 to 0.48; p=0.0001; Table 3) with the three-drug regimen relative to the two-drug regimen. An exploratory analysis according to the number of positive lymph nodes (pN1, 1-3 positive lymph nodes and pN2, 4-9 positive lymph nodes) was carried out. The three-drug regimen significantly decreased the risk of recurrence ( for pN1 patients, adjusted HR, 0.47; 95% CI, 0.24 to 0.90; *p*=0.023; for pN2 patients, adjusted HR, 0.26; 95% CI, 0.09 to 0.79; *p*=0.018; Table 4 and Table 5) and death (for pN1 patients, adjusted HR, 0.30; 95% CI, 0.12 to 0.74; *p*=0.009; for pN2 patients, adjusted HR, 0.10; 95% CI, 0.02 to 0.58; *p*=0.011; Table 4 and Table 5) both in pN1 and pN2 group compared with the two-drug regimen. Current study demonstrated patients with lymph node positive are likely to benefit from the taxane-based triplet chemotherapy compared with doublet chemotherapy in operable TNBC in adjuvant setting.

In the current study, the taxane-based three-drug regimen included a concurrent regimen (TAC) and a sequential regimen (AC-T). A 10-year analysis of the BCIRG-005 study in HER2-negative breast cancer with positive lymph node(s) showed that concurrent TAC was not superior in long-term DFS or OS effects to the sequential regimen (AC-T), and both regimens had comparable efficacy across all stratification subgroups 22. However, several studies have shown that the sequential regimen (AC-T) seems to be more effective than the concurrent regimen 4, 23, 24. Moreover, most of the guidelines recommend sequential regimens as preferred regimens in patients with high-risk breast cancers for both neoadjuvant and adjuvant settings. The current study did not find a significant difference in efficacy between the TAC regimen and the AC-T regimen in pN1-2 patients. Nevertheless, the AC-T regimen tended to decrease the risk of recurrence (adjusted HR, 0.61; 95% CI, 0.26 to 1.46; *p*=0.27; data not shown) and death (adjusted HR, 0.50; 95% CI, 0.13 to 2.01; *p*=0.329; data not shown) in patients with pN1-2 TNBC. Therefore, for this subgroup of breast cancer patients with positive lymph node sequential regimen is probably still the preferred regimen.

This study introduced lymph node stage into the selection of the taxane-based three-drug regimen and the two-drug regimen in operable TNBC in adjuvant setting, and found that the taxane-based three-drug regimen was not superior to the two-drug regimen in patients with negative lymph nodes, but was superior to the two-drug regimen in patients with one to nine positive lymph node(s). Our data suggested pathological lymph node status in patient with operable TNBC was likely to be more meaningful for selecting taxane-based two-drug regimen or three-drug regimen in adjuvant settings.

This study is a retrospective study with a small number of patients, especially for the subgroup analysis. As in patients with negative lymph node only 10.4% (23 of 222) events were reported, additional follow-up time and additional patients will be necessary to confirm the non-inferiority of the taxane-based two-drug regimen to three-drug regimen. In addition, whether AC-T regimen is superior to TAC regimen in patients with positive lymph node and which subgroup of patients with negative lymph nodes should be chemo-free after surgery still need more in-depth researches. However, this study focused on the operable TNBC and tried to find the most effective and least toxic regimen in adjuvant setting, and these results are useful for the clinical research and practice, especially for designing future trials on TNBC. Therefore, a randomized clinical trial will be necessary to identify whether lymph node stage guides taxane-based three-drug regimen and two-drug regimen selection in operable TNBC.

## Conclusion

The results from this retrospective study suggest that pathological lymph node stage leads to de-escalating chemotherapy strategies in patients with operable TNBC in adjuvant setting. Further research is required to identify more biomarkers or risk factors that can be used to optimize current adjuvant chemotherapy regimens in TNBC.

## Figures and Tables

**Table 1:** baseline patient and tumor characteristics according to pathological lymph node stage

		pN0			pN1-2		
		Three-drug regimen	Two-drug regimen	p#	Three-drug regimen	Two-drug regimen	*p*#
All cases		115 (%)	107 (%)		104(%)	55 (%)	
Age				0.28			0.74
	<=45	54 (47)	42 (39.2)		47 (45.2)	23 (41.8)	
	>45	61 (53)	65 (60.8)		57 (54.8)	32 (58.2)	
Histology				0.45			1.00
	Ductal	110 (95.6)	105 (98.1)		101 (97.1)	54 (98.2)	
	Others	5 (4.4)	2 (1.9)		3 (2.9)	1 (1.8)	
Clinical tumor stage				0.91			0.45
	T1	42 (36.5)	42 (49.3)		27 (26.0)	11 (20.0)	
	T2	70 (60.9)	62 (57.9)		72 (69.2)	39 (70.9)	
	T3	3 (2.6)	3 (2.8)		5 (4.8)	5 (9.1)	
Pathological tumor grade				0.05			0.73
	G1	3 (2.6)	8 (7.5)		3 (2.9)	2 (3.6)	
	G2	74 (64.3)	76 (71.0)		67 (64.4)	39 (70.9)	
	G3	33 (28.7)	19 (17.8)		32 (30.8)	14 (25.5)	
	missing	5 (4.4)	4 (3.7)		2 (1.9)	0	
Surgery				0.74			0.77
	Mastectomy	91 (79.1)	87 (81.3)		96 (92.3)	54 (98.2)	
	Breast conservative	24 (20.9)	20 (18.7)		8 (7.7)	1 (7.8)	

Three-drug regimen includes TAC and AC-T regimens; Two-drug regimen includes TA and TC regimens. TAC= taxane, anthracycline, and cyclophosphamide. AC-T= anthracycline and cyclophosphamide followed by a taxane. TA= taxane and anthracycline. TC= taxane and cyclophosphamide. # by Fisher's exact test

**Table 2:** First Disease-Free-Survival Event by Treatment Groups

			pN0			pN1-2	
			Three-drug regimen	Two-drug regimen		Three-drug regimen	Two-drug regimen
All cases			115 (%)	107 (%)		104 (%)	54 (%)
any event			11	12		23	30
breast cancer relapse							
	Locoregional		3	1		7	7
	Distant		8	8		15	22
Breast cancer			0	2		1	1
Second primary malignancy			0	1		0	0

The three-drug regimen includes the TAC and AC-T regimens, and the two-drug regimen includes the TA and TC regimens. TAC= taxane, anthracycline, and cyclophosphamide. AC-T= anthracycline and cyclophosphamide followed by a taxane. TA= taxane and anthracycline. TC= taxane and cyclophosphamide.

**Table 3:** Multivariable analysis of factors affecting survival in triple-negative breast cancer with pathological one to nine positive lymph node(s) (N=159)

		Disease-free survival				Overall survival		
Parameter	Group	*p*	HR	95% CI		*p*	HR	95% CI
Age (years)	≤45/>45	0.40	0.78	0.44-1.39		0.94	0.97	0.46-2.04
Histology	Ductal/others	0.23	0.41	0.10-1.74		0.44	0.45	0.06-3.43
Pathological tumor grade	G1-2/G3	0.10	0.61	0.34-1.09		0.65	0.84	0.39-1.82
Clinical tumor stage	T1/T2-3	0.14	0.56	0.26-1.21		0.52	0.72	0.27-1.93
Treatment regimen	Three-/two-drug	0.0004	0.37	0.22-0.64		0.0001	0.22	0.10-0.48

The three-drug regimen includes the TAC and AC-T regimens, and the two-drug regimen includes the TA and TC regimens. TAC= taxane, anthracycline, and cyclophosphamide. AC-T= anthracycline and cyclophosphamide followed by a taxane. TA= taxane and anthracycline. TC= taxane and cyclophosphamide. HR, hazard ratio.

**Table 4:** Multivariable analysis of factors affecting survival in TNBC with pathological one to three positive lymph nodes (N=113)

		Disease-free survival				Overall survival		
Parameter	Group	*p*	HR	95% CI		*p*	HR	95% CI
Age (years)	≤45/>45	0.25	0.66	0.32-1.35		0.66	0.82	0.34-2.00
Histology	Ductal/others	0.01	0.14	0.03-0.64		0.25	0.28	0.03-3.40
Pathological tumor grade	G1-2/G3	0.11	0.56	0.28-1.13		0.64	0.80	0.32-2.01
Clinical tumor stage	T1/T2-3	0.11	0.48	0.19-1.17		0.39	0.62	0.21-1.86
Treatment regimen	Three-/two-drug	0.023	0.47	0.24-0.90		0.009	0.30	0.12-0.74

The three-drug regimen includes the TAC and AC-T regimens, and the two-drug regimen includes the TA and TC regimens. TAC= taxane, anthracycline, and cyclophosphamide. AC-T= anthracycline and cyclophosphamide followed by a taxane. TA= taxane and anthracycline. TC= taxane and cyclophosphamide. HR, hazard ratio.

**Table 5:** Multivariable analysis of factors affecting survival in TNBC with pathological four to nine positive lymph nodes (N=46)

		Disease-free survival				Overall survival		
Parameter	Group	*p*	HR	95% CI		*p*	HR	95% CI
Age (years)	≤45/>45	0.81	0.88	0.29-2.60		0.95	1.06	0.21-5.45
Histology	Ductal/others	0.99	-	-		0.99	-	-
Pathological tumor grade	G1-2/G3	0.46	0.66	0.22-1.99		0.89	0.89	0.18-4.36
Clinical tumor stage	T1/T2-3	0.90	1.11	0.21-5.76		0.49	2.35	0.21-26.48
Treatment regimen	Three-/two-drug	0.018	0.26	0.09-0.79		0.011	0.10	0.02-0.58

The three-drug regimen includes the TAC and AC-T regimens, and the two-drug regimen includes the TA and TC regimens. TAC= taxane, anthracycline, and cyclophosphamide. AC-T= anthracycline and cyclophosphamide followed by a taxane. TA= taxane and anthracycline. TC= taxane and cyclophosphamide. HR, hazard ratio.
